# Value of the lung immune prognostic index in patients with advanced small cell lung cancer treated with programmed death-ligand 1 and programmed death-1 inhibitors in the Chinese alpine region

**DOI:** 10.3389/fonc.2024.1411548

**Published:** 2024-09-26

**Authors:** Meiling Zhang, Jingwei Hao, Yunjiao Wu, Ziyi Gao, Meng Wang

**Affiliations:** Department of Respiratory Medical Oncology, Harbin Medical University Cancer Hospital, Harbin, Heilongjiang, China

**Keywords:** lung immune prognostic index (LIPI), small cell lung cancer (SCLC), programmed death-ligand 1 (PD-L1), programmed death-1 (PD-1), immune-related adverse events (irAEs), prognostic factor

## Abstract

**Purpose:**

To assess the potential added value of the lung immune prognostic index (LIPI) in patients with small cell lung cancer (SCLC), treated with programmed death-ligand 1 (PD-L1)/programmed death-1 (PD-1) inhibitors, who lived in the Chinese alpine region.

**Methods:**

120 SCLC patients treated with PD-L1/PD-1 inhibitors were divided into three LIPI groups, from July 2018 to April 2021. Cox regression models were used to evaluate the prognostic effect of three LIPI groups on overall survival (OS) and progression-free survival (PFS). Logistic regression analysis was conducted to explore the association between immune-related adverse events (irAEs) and the pretreatment of neutrophil-to-lymphocyte ratio (dNLR), lactate dehydrogenase (LDH), and LIPI.

**Results:**

The median OS was 4.5, 6.3, and 10.0 months (*p=0.001*) and the median PFS was 2.5, 4.3, and 5.3 months (*p=0.049*) for Poor, Intermediate, and Good LIPI, respectively. The disease control rate (DCR) was also higher in the Good LIPI group (*p=0.003*). Moreover, multivariate analysis confirmed that worse LIPI was correlated with shorter OS and PFS. dNLR was associated with the onset of irAEs, not LIPI. Conclusion: The LIPI might be a promising predictive and prognostic biomarker in SCLC patients treated with PD-L1/PD-1 inhibitors in the Chinese Alpine region.

## 1 Introduction

Globally, lung cancer is the second most commonly diagnosed cancer and remains the leading cause of cancer-related death (1).In China, lung cancer is the most common cancer which is the leading cause of new cases and deaths (2). Small cell lung cancer (SCLC) is a highly aggressive neuroendocrine tumor, which is strongly associated with smoking and accounts for approximately 15% of all lung cancers and 7% five-year survival (3). The preferred regimen for SCLC patients is platinum-based doublet chemotherapy, and the etoposide plus platinum (EP) combination has been the standard first-line treatment of SCLC for many years (4). Although SCLC is highly sensitive to chemoradiotherapy in the early stages, most patients develop tumor progression or recurrence due to resistance. Encouragingly, the application of immunotherapy has revolutionized the therapeutic paradigm of SCLC in recent years.

Immunotherapy, particularly immune checkpoint inhibitors (ICIs), plays an important role in the treatment of SCLC. The combination of PD-L1 and EP in the treatment of SCLC was proposed for the first time in 2019, and the addition of PD-L1 can increase the survival rate by 3 times (5). Several large clinical trials indicated SCLC patients treated with PD-L1/PD-1 inhibitors could benefit with respect to progression free survival (PFS) or overall survival (OS) (6–8). However, the benefit with PD-L1/PD-1 inhibitors is not seen in all SCLC patients. With or without ICIs, the response rate of first-line treatment for SCLC patients remains at 60%-65% (9).

Currently, it is known that PD-L1 expression is one of the most frequently discussed biomarkers and is used to guide the clinical efficacy of PD-L1/PD-1 inhibitors. Although NCCN guidelines indicate that PD-L1 expression can predict the efficacy of immunotherapy, the level of PD-L1 expression in SCLC is likely to be an unreliable predictive biomarker for ICIs. Many clinical trials have confirmed there was no significant correlation between the expression of PD-L1 and ICIs efficiency in SCLC patients (6, 7), including many cases with PD-L1 positive tumors. Hellmann et al. found the tumor mutational burden (TMB) might be a potential biomarker for identifying SCLC patients who tended to respond to ICIs (10). Nevertheless, the disadvantage is that the TMB doesn’t have a clear cutoff value. Therefore, there is an urgent need to determine an appropriate biomarker to identify the beneficial population with PD-L1/PD-1 inhibitor treatment in SCLC patients.

Systemic inflammation is closely associated with disease progression of most tumors, including lung cancer (11). Over the past few years, several studies have affirmed the predictive value of various inflammatory biomarkers in cancer, such as neutrophil-lymphocyte ratio (NLR) and platelet-lymphocyte ratio(PRL) (12, 13).In 2018, Mezquita et al. described a novel biomarker, the lung immune prognostic index (LIPI), which integrates baseline derived neutrophil-to-lymphocyte ratio (dNLR) and lactate dehydrogenase (LDH) (14). Then, the relationship between the LIPI and survival prognosis in patients was reported in some solid tumors, such as metastatic urothelial carcinoma (mUC) (15)and hepatocellular carcinoma (HCC) (16). The research has also shown that there is a strong correlation between LIPI and early death under the new invasive progression model (17). LIPI has more advantages than other inflammatory biomarkers.

Based on the above, we hypothesize that there is a correlation between LIPI and the response to treatment in SCLC patients. In this study, for the first time, LIPI was used to explore the prognosis of SCLC patients treated with PD1/PD-L1 inhibitors in the Chinese Alpine region.

## 2 Methods

### 2.1 Patients

We conducted a retrospective study in a cohort of 120 patients with SCLC receiving PD-L1/PD-1 inhibitor therapy in the Chinese Alpine region. The inclusion criteria of SCLC patients at the Harbin Medical University Cancer Hospital treated with PD-L1/PD-1 inhibitors therapy from July 2018 to April 2021 were as follows: (1) patients who were diagnosed with SCLC by histopathology or cytopathology; (2) at least one cycle of PD-L1/PD-1 inhibitor therapy, either monotherapy or combination therapy; (3) patients in whom the complete blood cell counts and LDH level were obtained at baseline before ICI therapy. Then, basic clinical and pathological data were collected from patients who met these criteria. Missing values regarding characteristics and tumor response were ignored.

### 2.2 Evaluation

Imaging examination was performed every 8–12 weeks, and tumor responses were assessed by two clinical oncologists independently according to the Response Evaluation Criteria in Solid Tumors guidelines version 1.1 (RECIST1.1). Overall survival (OS) was defined as the time from the start of treatment with PD-L1/PD-1 inhibitors to death. Progression free survival (PFS) was defined as the time from the start of treatment with PD-L1/PD-1 inhibitors to disease progression. The disease control rate (DCR) was defined as complete plus partial response plus stable disease, and the overall response rate (ORR) was defined as complete plus partial response.

### 2.3 The LIPI

The LIPI is a marker that combines the level of dNLR and serum LDH. The LIPI was calculated, as previously described by Mezquita et al (14). The calculation formula of dNLR was as follows: absolute neutrophil count (white blood cell count - absolute neutrophil count). Patients were divided into three LIPI groups according to the cutoff value of dNLR and the upper limit of normal range of LDH before treatment. Afterward, patients with dNLR greater than 3 (high-dNLR) and LDH higher than the upper limit of normal (ULN) (high-LDH) were categorized into the “Poor LIPI group,” patients with dNLR greater than 3 and LDH lower than ULN or dNLR less than 3 and LDH higher than ULN were categorized into the “Intermediate LIPI group,” and patients with dNLR less than 3 (low-dNLR) and LDH lower than ULN (low-LDH) were categorized into the “Good LIPI group”.

### 2.4 Statistical analysis

Statistical analyses were performed using IBM SPSS Statistic 25.0. The chi-squared or Fisher’s exact test was used to compare clinicopathological data among the Poor, Intermediate, and Good LIPI groups. Continuous variables were compared using the t-test. Survival analyses were performed using the Kaplan–Meier method and the log-rank test. Univariate and multivariate Cox regression analyses were used to investigate the prognostic factors associated with OS and PFS. The impact of the baseline LIPI on the DCR and ORR was represented by a column diagram. Logistic regression models were used to analyze the correlation between peripheral blood markers and the onset of irAEs. A *p* value < 0.05 was considered statistically significant.

## 3 Results

### 3.1 Patient clinical characteristics and outcomes

**Table 1** summarizes the clinical characteristics and outcomes of the patients. A total of 120 patients across the Chinese Alpine region were enrolled in this study. Further, 48.3% (n=58) of the patients had good LIPI, while 42.5% (n=51) and 9.2% (n=11) had intermediate and poor LIPI, respectively. Eastern Cooperative Oncology Group Performance Status (ECOG PS) was 0 in 12 patients (10.0%), 1 in 87 patients (72.5%), and 2 in 21 patients (17.5%). Extended disease accounted for the majority of cases (94.2%). Moreover, 31.7% (n=38) of patients received first-line therapy, 30.8% (n=37) received second-line therapy, and 36.5% (n=45) received third-line and above therapies. Patients receiving PD-L1 inhibitors accounted for 60.0% (n=72) of the cases. Further, 99 patients (82.5%) received ICIs combined with chemotherapy, and the remaining 21 patients (17.5%) received ICIs as monotherapy. A total of 22.5% of patients had brain metastases, 35.8% had liver metastases, 41.7% had bone metastases, 45.0% had pleural or pericardial metastases, and 14.2% had adrenal metastases.

**Table 1:** Clinical characteristics of the 120 patients treated with PD-L1/PD-1 inhibitors.

Clinical characteristics	Total [n (%)]	Good LIPI [n (%)]	Intermediate LIPI [n (%)]	Poor LIPI [n (%)]	*p* value
Total	120 (100)	58 (48.3)	51 (42.5)	11 (9.2)	
Age					0.895
≤60	61 (50.8)	29 (50.0)	27 (52.9)	5 (45.5)	
>60	59 (49.2)	29 (50.0)	24 (47.1)	6 (54.5)	
Gender					0.360
Male	74 (61.7)	32 (55.2)	34 (66.7)	8 (72.7)	
Female	46 (38.3)	26 (44.8)	17 (33.3)	3 (27.3)	
Smoking status					0.929
Current	60 (50.0)	30 (51.8)	25 (49.0)	5 (45.5)	
Former	27 (22.5)	14 (24.1)	11 (21.6)	2 (18.2)	
Never	33 (27.5)	14 (24.1)	15 (29.4)	4 (36.3)	
ECOG-PS					0.084
0	12 (10.0)	7 (12.1)	5 (9.80)	0 (0)	
1	87 (72.5)	36 (62.0)	40 (78.4)	11 (100)	
2	21 (17.5)	15 (25.9)	6 (11.8)	0 (0)	
Stage					0.849
Extended disease	113 (94.2)	55 (94.8)	47 (92.2)	11 (100)	
Limited disease	7 (5.8)	3 (5.2)	4 (7.8)	0 (0)	
Therapy line					0.327
1	38 (31.7)	18 (31.0)	19 (37.3)	1 (9.1)	
2	37 (30.8)	18 (31.0)	15 (29.4)	4 (36.4)	
≥3	45 (36.5)	22 (38.0)	17 (33.3)	6 (54.5)	
Immunotherapy drug					0.826
PD-L1	72 (60.0)	34 (58.6)	32 (62.7)	6 (54.5)	
PD-1	48 (40.0)	24 (41.4)	19 (37.3)	5 (45.5)	
Regimen					0.722
Combination therapy	99 (82.5)	46 (79.3)	43 (84.3)	10 (90.9)	
Monotherapy	21 (17.5)	12 (20.7)	8 (15.7)	1 (9.1)	
Brain metastases					0.144
Yes	27 (22.5)	9 (15.5)	16 (31.4)	2 (18.2)	
No	93 (77.5)	49 (84.5)	35 (68.6)	9 (81.8)	
Liver metastases					0.131
Yes	43 (35.8)	18 (31.0)	18 (35.3)	7 (63.6)	
No	77 (64.2)	40 (69.0)	33 (64.7)	4 (36.4)	
Bone metastases					0.043
Yes	50 (41.7)	31 (53.4)	16 (31.4)	3 (27.3)	
No	70 (58.3)	27 (46.6)	35 (68.6)	8 (72.7)	
Pleural or pericardial metastases					0.662
Yes	54 (45.0)	27 (46.6)	21 (41.2)	6 (54.5)	
No	66 (55.0)	31 (53.4)	30 (58.8)	5 (45.5)	
Adrenal metastases					0.465
Yes	17 (14.2)	10 (17.2)	7 (13.7)	0 (0)	
No	103 (85.8)	48 (82.8)	44 (86.3)	11 (100)	

PD-L1/PD-1, programmed death-ligand 1/programmed death-1; LIPI, lung immune prognostic index; ECOG-PS, Eastern Cooperative Oncology Group Performance Status.

### 3.2 Association between the LIPI and the prognostic utility of SCLC patients treated with ICIs

**Figure 1:**
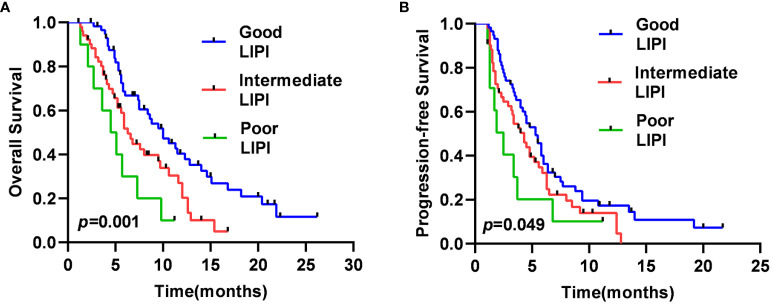
Kaplan–Meier survival curves for SCLC patients of OS **(A)** and PFS **(B)** in different LIPI groups.

The median OS was 4.5 months [95% confidence interval (CI), 2.1–6.8 months], 6.3 months (95% CI, 5.1–7.5 months), and 10.0 months (95% CI, 7.2–12.8 months) in the Poor, Intermediate, and Good LIPI groups, respectively (*p=0.001*) (**Figure 1A**). The median PFS was 2.5 months (95% CI, 1.3–3.7 months), 4.3 months (95% CI, 3.1–5.4 months), and 5.3 months (95% CI, 4.3–6.2 months) in the Poor, Intermediate, and Good LIPI groups, respectively (*p=0.049*) (**Figure 1B**). These results suggested that OS and PFS in the Good LIPI group were significantly higher than those in the Poor or Intermediate LIPI group.

### 3.3 Association between the LIPI and the predictive utility of SCLC patients treated with ICIs

**Figure 2:**
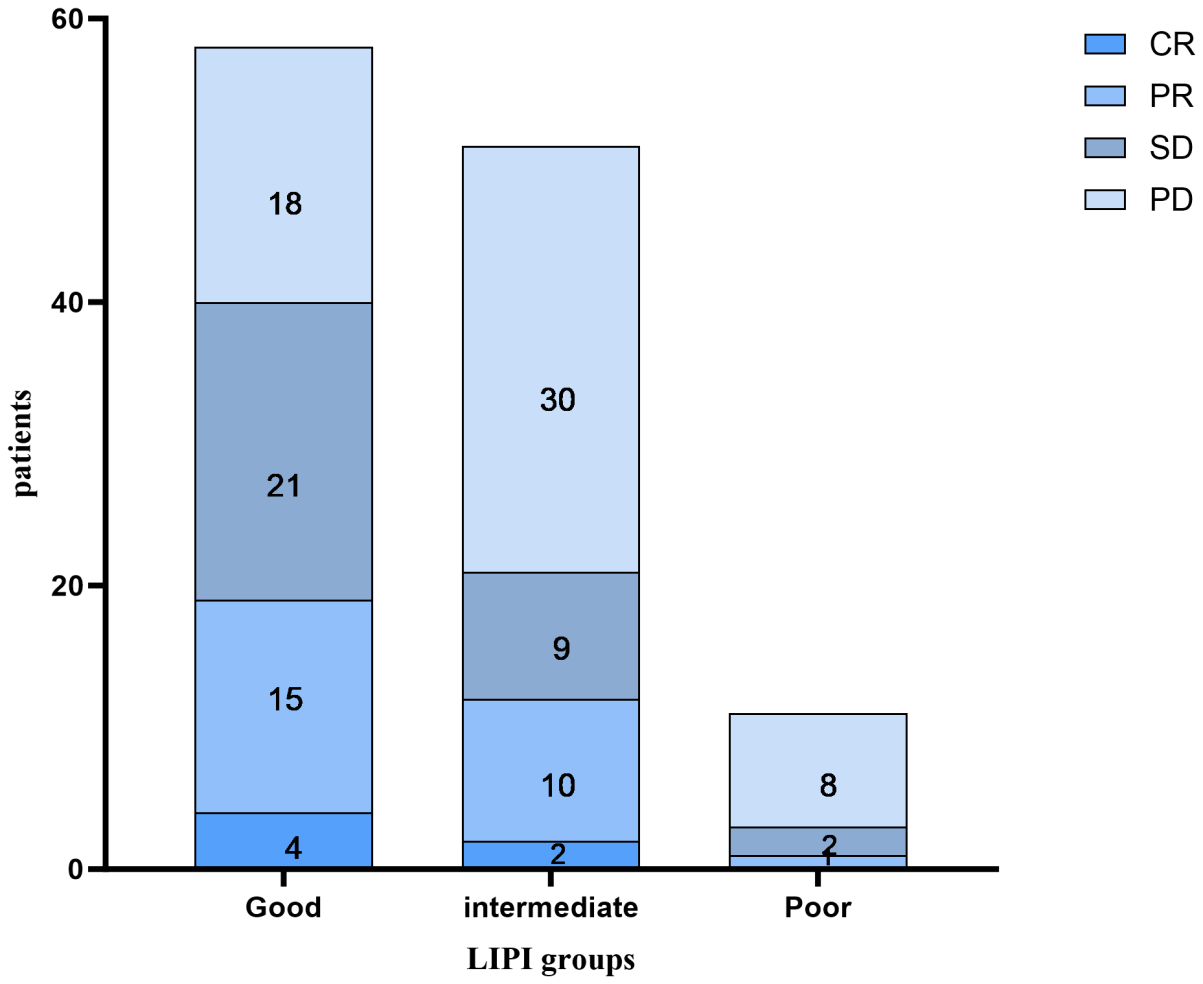
The distribution between the response to immune checkpoint inhibitors and the LIPI groups. CR complete response, PR partial response, SD stable disease, PD progressive disease.

As shown in **Figure 2**, of the 58 patients in the Good LIPI group, 4 experienced a complete response (CR), 15 experienced a partial response (PR), 21 experienced stable disease (SD), and 18 experienced progressive disease (PD). Among the 51 patients in the Intermediate LIPI group, 2 experienced a CR, 10 experienced a PR, 9 experienced SD, and 30 experienced PD. Among the 11 patients in the Poor LIPI group, 1 experienced a PR, 2 experienced SD, and 8 experienced PD. The DCRs in the Poor, Intermediate, and Good LIPI groups were 27.3%, 41.2%, and 69.0%, respectively (*p=0.003*) (**Table 2**). The ORRs in the Poor, Intermediate, and Good LIPI groups were 9.1%, 23.5%, and 32.8%, respectively (*p=0.225*) (**Table 2**). These results indicated that the DCR was higher in the Good LIPI group than the Poor or Intermediate LIPI group; however, there was no significant correlation between the LIPI and ORR.

**Table 2:** The relationship between clinical response and LIPI groups in SCLC patients treated with PD-L1/PD-1 inhibitors.

Indicator		All patients [N=120, n (%)]	LIPI Group [n (%)]			*p* value
			Good (N=58)	Intermediate (N=51)	Poor (N=11)	
Response rate	CR	6 (5.0)	4 (6.9)	2 (3.9)	0 (0)	0.051
	PR	26 (21.7)	15 (25.9)	10 (19.6)	1 (9.1)	
	SD	32 (26.7)	21 (36.2)	9 (17.7)	2 (18.2)	
	PD	56 (46.6)	18 (31.0)	30 (58.8)	8 (72.7)	
DCR	CR, PR or SD	64 (53.4)	40 (69.0)	21 (41.2)	3 (27.3)	0.003
ORR	CR or PR	32 (26.7)	19 (32.8)	12 (23.5)	1 (9.1)	0.225

LIPI, lung immune prognostic index; SCLC, small cell lung cancer; CR, complete response; PR, partial response; SD, stable disease; PD, progressive disease; DCR, disease control rate; ORR, overall response rate.

### 3.4 Univariate and multivariate analyses of OS and PFS

**Figure 3:**
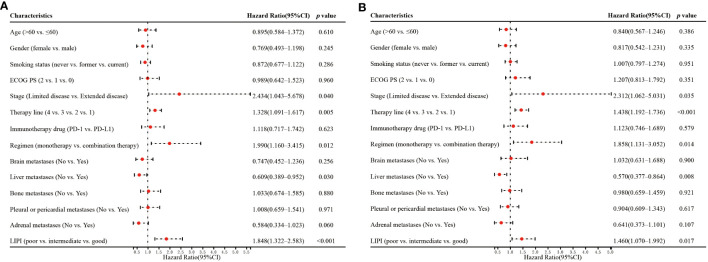
Univariate analysis of factors associated with OS **(A)** and PFS **(B)**.

**Figure 4:**
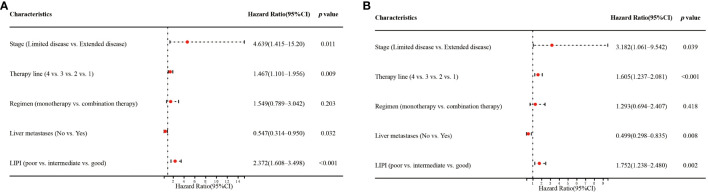
Multivariate analysis of factors associated with OS **(A)** and PFS **(B)**.

Univariate analysis showed that stage (*p=0.040*), therapy line (*p=0.005*), regimen (*p=0.012*), liver metastases (*p=0.030*), and LIPI (*p<0.001*) significantly affected OS (**Figure 3A**). In the multivariate analysis, worse LIPI was significantly associated with shorter OS [hazard ratio (HR)=2.372; 95% CI: 1.608–3.498; *p<0.001*] (**Figure 4A**). These results suggested that LIPI was an independent prognostic factor for OS.

Similarly, univariate analysis showed that stage (*p=0.035*), therapy line (*p<0.001*), regimen (*p=0.014*), liver metastases (*p=0.008*), and LIPI (*p=0.017*) significantly affected PFS (**Figure 3B**). In the multivariate analysis, worse LIPI was significantly related to shorter PFS (HR=1.752; 95% CI: 1.238–2.480; *p=0.002*) (**Figure 4B**). These results indicated that LIPI was an independent prognostic factor for PFS.

### 3.5 Immune-related adverse events

**Figure 5:**
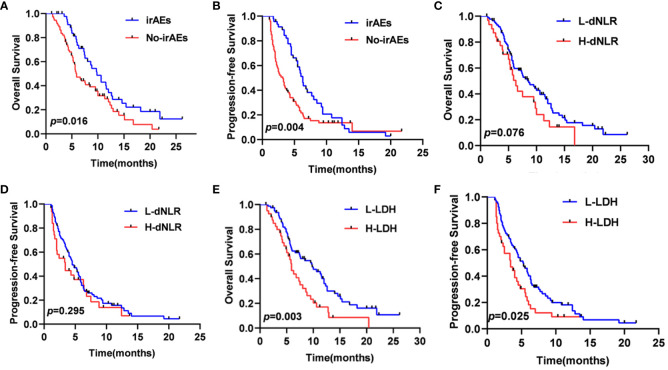
OS **(A)** and PFS **(B)** curves of patients according to the onset of irAEs, OS **(C)** and PFS **(D)** curves of patients according to the dNLR, OS **(E)** and PFS **(F)** curves of patients according to the LDH.

In total, 46 patients (38.3%) experienced six different irAEs of any grade, which included rash (n=17, 37.0%), liver dysfunction (n=10, 21.7%), hypothyroidism (n=8, 17.4%), diarrhea (n=7, 15.2%), infusion reaction (n=3, 6.5%), and impaired glucose regulation (n=1, 2.2%). The most common severe irAEs (grade≥3) were rash (n=4, 8.7%). The median OS of the 46 patients with irAEs (9.8 months, 95% CI: 7.5–12.0 months) was significantly better than that of the 74 patients without irAEs (5.9 months, 95% CI: 4.7–7.0 months). Similarly, the median PFS of the patients with irAEs (6.3 months, 95% CI:5.5–7.0 months) was significantly preferable to that of patients without irAEs (3.2 months, 95% CI: 2.3–4.0 months) (**Figures 5A, B**). It also showed that the patients with lower LDH significantly had better median OS and PFS (*p=0.003, p=0.025*) (**Figures 5E, F**). However, the patients with lower dNLR did not show longer median OS and PFS (*p=0.076, p=0.295*) (**Figures 5C, D**).

The low-dNLR, low-LDH, Good LIPI, and Intermediate LIPI groups were composed of 41 (46.6%), 31 (39.2%), 28 (48.3%), and 16 (31.4%) patients, respectively (**Table 3**). The univariate and multivariate logistic regression analyses indicated an association between low-dNLR and any grade of irAEs (*p=0.004, p=0.035*). However, the LIPI was not an independent predictor of the onset of irAEs in the multivariate analysis (*p=0.675, p=0.936*).

**Table 3:** Levels of the peripheral blood markers by irAE development.

Blood marker	I r AEs (n (%))	Univariate	*p* value	Multivariate	*p* value
		OR (95% CI)		OR (95% CI)	
L-d NLR (n=88)	41 (46.6)	4.711 (1.661–13.356)	0.004	4.588 (1.109–18.975)	0.035
H-d NLR (n=32)	5 (15.6)	1		1	
L-LDH (n=79)	31 (39.2)	1.119 (0.513–2.441)	0.777	0.750 (0.106–5.324)	0.774
H-LDH (n=41)	15 (36.6)	1		1	
Good LIPI (n=58)	28 (48.3)	4.200 (0.834–21.147)	0.082	1.627 (0.189–13.989)	0.657
Intermediate LIPI (n=51)	16 (31.4)	2.057 (0.398–10.630)	0.389	0.915 (0.106–7.869)	0.936
Poor LIPI (n=11)	2 (18.2)	1		1	

OR, odds ratio; CI, confidence interval; irAEs, immune-related adverse events; dNLR, derived neutrophil-to-lymphocyte ratio; LDH, lactate dehydrogenase; LIPI, lung immune prognostic index; L-dNLR, low-dNLR; L-LDH, low-LDH; H-dNLR, high-dNLR; H-LDH, high-LDH.

## 4 Discussion

In recent years, although ICIs have been successfully applied in SCLC patients, not all patients benefit from it. There are still challenges in identifying biomarkers which are effective, reliable and easy for SCLC patients treated with ICIs. Currently, more focus has been turned toward the correlation between inflammation and the clinical outcomes of Tumor patients who are undergoing PD-L1/PD-1 inhibitors treatment. The Inflammatory process has been confirmed to be closely related to the immune resistance in patients with cancer, which promotes cancer growth and dissemination, and it can activate a variety of oncogenic signaling pathways (18). Previous studies have shown that a higher NLR was associated with poor prognosis in a variety of cancers, including head and neck cancer, lung cancer, RCC, and breast cancer (17, 19–21). It is known to all that dNLR is superior to NLR. Similarly, more studies have found that a higher dNLR was correlated with worse prognosis (14, 22–24). LDH is also a classic inflammatory indicator in tumor patients, and it is a glycolytic enzyme that can be released by rapidly growing tumors. Some studies have shown that there was a correlation between LDH and the prognosis of tumors, including lung cancer (12, 14, 25, 26). Therefore, patients with a higher dNLR and LDH are more likely to accelerate tumor progression, and the specific underlying mechanisms need to be studied further. The study revealed that there were statistical differences in median OS and PFS among the Good, Intermediate, and Poor LIPI groups of melanoma patients receiving immunotherapy (*p<0.0001*). Moreover, the Intermediate and Poor LIPI groups were significantly associated with worse prognosis compared to the Good LIPI group in a multivariate analysis, with an HR of 3.3 (95% CI, 2.0–5.3; *p<0.001*) and 7.9 (95% CI, 4.1–15.2; *p<0.001*), respectively (27)However, it is not clear whether LIPI can be used as a strong prognostic factor for patients with SCLC. The purpose of this study was to verify the predictive value of LIPI on survival rate, remission rate and irAEs of patients with SCLC treated with ICIs in the Chinese alpine region.

In this study, retrospective analysis was used to collect patients with SCLC in the Chinese alpine region. As far as we know, tobacco and PM2.5 are unique regional differences in the Chinese alpine region, and they are also common causes of high incidence of lung cancer (28). As shown in **Table 1** of this research, about 75% of people have smoking history. Our study found LIPI was significantly relevant to the DCR in these patients. There was a higher proportion of disease progression in the Intermediate and Poor LIPI groups than in the Good LIPI group. At the same time, research showed that preoperative LIPI could predict the prognosis of patients with bladder cancer (BC) undergoing radical cystectomy (29). Inflammation was able to induce or promote tumor formation or metastasis through the tumor microenvironment. In the tumor microenvironment, rapid generation of neutrophils might lead to massive release of immature or poorly differentiated neutrophils in the pro-inflammatory state with tumor-promoting effects (30, 31), in addition, the metabolic competition between T cells and tumor cells weakens the efficacy of PD-L1/PD-1 inhibitors (32). We also found multivariate analysis indicated that the LIPI was an independent prognostic factor for PFS and OS in SCLC patients through ICIs therapy. Therefore, LIPI can be used as an Inflammatory biomarker for patients with SCLC who were treated with ICIs in the Chinese alpine area and can be used to evaluate the survival rate and remission rate of patients of SCLC.

In addition, with the growing use of PD-L1/PD-1 inhibitors, irAEs also increased, which may cause treatment disruption and even death. The present study also explored the relationship between irAEs and the peripheral blood markers—dNLR, LDH, and LIPI; it also found only low-dNLR was significantly associated with the occurrence of irAEs. The study demonstrated the advanced esophageal cancer with irAEs had superior outcomes from ICIs, including better PFS and OS (33). Seiwert et al. also explored the association between development of irAEs and prolonged OS in patients with head and neck cancer receiving ICIs (34). Simultaneously, our current research showed that the patients who experienced the relevant irAEs had longer OS and PFS. Overall, the results of this study, as well as others, showed that the LIPI was correlated with the efficacy and outcome of SCLC patients through ICIs therapy. However, the pretreatment LIPI may not be used as a convenient marker to distinguish irAEs in a timely manner.

This study shows that LIPI is a promising biomarker in SCLC patients treated with PD-L1/PD-1 inhibitors and it can be used to evaluate the efficacy and prognosis. Although the sample size of this study is small and there may be a deviation, the prediction and application value of this study in clinical practice should not be underestimated. In brief, LIPI is a simple and readily available global tool used to evaluate prognostic value in prospective studies with large tumor samples.

## 5 Conclusions

The LIPI based on dNLR and LDH is an independent prognostic factor for PFS and OS and is significantly relevant to the DCR. However, the LIPI is not an independent predictor of the occurrence of irAEs. LIPI appears to be a promising predictive and prognostic indicator in SCLC patients in the Chinese alpine region during ICIs treatment. Before ICIs treatment, the calculation of LIPI index in patients with SCLC may avoid or reduce the occurrence of irAEs and improve the prognosis.

## Data availability statement

The original contributions presented in the study are included in the article/supplementary material. Further inquiries can be directed to the corresponding author.

## Ethics statement

The studies involving humans were approved by the Institutional Review Board of the Harbin Medical University Cancer Hospital. The studies were conducted in accordance with the local legislation and institutional requirements. The participants provided their written informed consent to participate in this study. Written informed consent was obtained from the individual(s) for the publication of any potentially identifiable images or data included in this article.

## Author contributions

MZ: Data curation, Methodology, Investigation, Writing – original draft, Writing – review & editing. JH: Data curation, Resources, Formal analysis, Writing – original draft. YW: Data curation, Resources, Formal analysis, Writing – original draft. ZG: Software, Writing – original draft. MW: Visualization, Supervision, Conceptualization, Funding acquisition, Project administration, Writing – review & editing.
